# Medical student mistreatment: an inconvenient truth?

**DOI:** 10.1080/10872981.2019.1633172

**Published:** 2019-06-20

**Authors:** Mohammed Allaf, Omer Mohamed, Hussein Elghazaly

**Affiliations:** Imperial College London

Letter to the editor,

We read with great interest the study by Markman et al [1] regarding medical student mistreatment and humiliation. Their finding that 55% of students experienced public humiliation is surprising as this is more common than previously cited [2]. As 5^th^ year medical students at Imperial College School of Medicine, we wonder whether this is seen commonly in other medical schools.

We appreciate their efforts in conducting this study; however, there are a few factors which need addressing. First, it seems that recruitment for this study proved difficult as only 29 (28 third-year and 1 fourth-year) students were involved. This number is too small to draw generalisable conclusions about the experience of public humiliation in US medical schools. In order to address this, incentivized participation could work. This could include giving monetary reward or discount vouchers to those who attend. Regarding this, selection bias is a notable consideration. As enrolment was optional, students participating in focus groups may have been exposed to more negative experiences on wards compared to students who do not participate. We wonder whether an online anonymous questionnaire would yield greater participation with students with a mixture of different experiences. This was seen in a study by Tim et al which saw a much larger participation of 309 medical and nursing students [3].

Second, the very nature of extracting information in a focus group may question the reliability of the data. Discussion in a focus group setting may have influenced the responses of the participants. Students may confer to conformational bias and reiterate other participants’ answers. Additionally, the moderators of the focus group were study investigators of the clinical rotations the students were undertaking. This could have influenced student responses. We suggest that one-to-one interviews with an external investigator may encourage students to give a more accurate representation of their experiences.

Furthermore, this study does not explore in depth the barriers to reporting mistreatment. Chung et al suggest that reporting mistreatment is low due to a myriad of reasons including fear of reprisal, perception of medical culture as well as the difficult logistics of reporting [4]. This study may benefit from more questions focusing on whether these experiences are not being reported due to fear of reprisal or simply due to the difficult logistical nature of reporting.

The findings of this paper are critical, but further understanding of the extent of mistreatment is crucial. Rautio et al report that mistreatment is most common amongst medical students compared to other university students and more common amongst females [5]. The prevalence of public humiliation found by this study is far greater than previously reported [2]. We wonder whether differences in baseline characteristics may have accounted for this. Subgroup analyses of gender and current specialty rotation may provide a better insight into this problem. Further research is needed to better understand this mistreatment and the interventions needed to reduce it.
